# Inverse-Designed Ultra-Compact Passive Phase Shifters for High-Performance Beam Steering

**DOI:** 10.3390/s24217055

**Published:** 2024-11-01

**Authors:** Tianyang Fu, Mengfan Chu, Ke Jin, Honghan Sha, Xin Yan, Xueguang Yuan, Yang’an Zhang, Jinnan Zhang, Xia Zhang

**Affiliations:** 1 State Key Laboratory of Information Photonics and Optical Communications, Beijing University of Posts and Telecommunications, Beijing 100876, China; futianyang@ime.ac.cn (T.F.); cmf@bupt.edu.cn (M.C.); kjin@bupt.edu.com (K.J.); shahonghan@bupt.edu.cn (H.S.); yuanxg@bupt.edu.cn (X.Y.); zhang@bupt.edu.cn (Y.Z.); zhangjinnan@bupt.edu.cn (J.Z.); xzhang@bupt.edu.cn (X.Z.); 2 Institute of Microelectronics, Chinese Academy of Sciences, Beijing 100029, China

**Keywords:** inverse design, phase shifter, beam steering

## Abstract

Ultra-compact passive phase shifters are inversely designed by the multi-objective particle swarm optimization algorithm. The wavelength-dependent phase difference between two output beams originates from the different distances of the input light passing through the 4 μm × 3.2 μm rectangular waveguide with random-distributed air-hole arrays. As the wavelength changes from 1535 to 1565 nm, a phase difference tuning range of 6.26 rad and 6.95 rad is obtained for TE and TM modes, respectively. Compared with the array waveguide grating counterpart, the phase shifters exhibit higher transmission with a much smaller footprint. By combining the inverse-designed phase shifter and random-grating emitter together, integrated beam-steering structures are built, which show a large scanning range of ±25.47° and ±27.85° in the lateral direction for TE and TM mode, respectively. This work may pave the way for the development of ultra-compact high-performance optical phased array LiDARs.

## 1. Introduction

Optical phased array (OPA) has attracted considerable attention in Light Detection and Ranging (LiDAR) due to the advantages of small size, high scanning accuracy, excellent directionality, and fast scanning speed. The phase shifter is a critical device for OPA, which plays an important role in scanning angle and operation speed. Currently, the most popular phase shifter used in OPAs is the thermal–optic phase shifter [1,2,3,4,5,6], which is facing the challenges of slow speed, additional fabrication process, and thermal crosstalk among waveguides. The electro-optic phase shifter has a faster speed, but is limited by the high loss caused by doping as well as the complex design [7,8]. Another kind of phase shifter with potential is the passive phase shifter, which generates phase delay by simply extending waveguides [9,10,11]. Passive phase shifters are generally in the form of array waveguide grating (AWG), snakelike geometry, or imbalanced tree architecture, among which AWG is the most popular structure. Take AWG as an example. Light enters different waveguides separately, and the length of waveguides has a linear gradient. As a result, the phase in different waveguides has a linear gradient, resulting in a phase difference between adjacent waveguides [12]. Although passive phase shifters can overcome the disadvantages of thermal–optic and electronic–optic phase shifters, the relatively large footprint and phase errors limit their applications in ultra-compact high-performance OPAs.

In recent years, inverse design has gained increasing attention due to its potential in the design of ultra-compact high-performance optical devices. This approach combines an optimization algorithm with electromagnetic calculation, which is able to find optimal structures in infinite parameter space to reach the design goal. Based on this method, a great deal of optical devices, such as polarization beam splitters [13], optical logical gates [14], optical varifocal metalens [15], and optical waveguide crossings [16], have been demonstrated, which exhibited excellent performance with a rather small footprint. The inverse design of OPAs has been reported in recent years, including sparse aperiodic arrays designed by particle swarm optimization [17], non-uniform sparse OPA designed by genetic algorithm [18], photonic crystal circuits for optical beam steering [19], etc. Up till now, inverse design studies on optical power splitters using active phase shifters [20] and passive phase shifters [21,22] have been reported. However, there is no research combining inverse-designed phase shifters and beam-steering structures.

In this work, the inverse design method is used to design ultra-compact passive phase shifters and integrated beam-steering structures. By introducing air-hole arrays into a 4 μm × 3.2 μm rectangular waveguide, both TE and TM phase shifters are obtained. The multi-objective particle swarm optimization (MOPSO) algorithm is used to optimize the distribution of air holes to achieve the optimized performance. As the wavelength changes from 1535 to 1565 nm, a phase difference tuning range of 6.26 rad and 6.95 rad is obtained for the TE and TM modes, respectively. It also exhibits high transmission and a small footprint, significantly superior to its AWG counterpart with a much larger footprint. Moreover, an integrated beam-steering structure is built by combining the inverse-designed phase shifter and random-grating emitter together, which shows a large scanning range of ±25.47° and ±27.85° for the TE and TM modes, respectively. The inverse-designed phase shifters show great potential in ultra-compact high-performance OPAs.

## 2. Materials and Methods

The passive phase shifters are inversely designed by the MOPSO algorithm. Optimization problems with multiple (usually conflicting) objectives are called multi-objective optimization problems (MOPs) [23], which are bound to simultaneously optimize multiple objectives [24]. Moore and Chapman first used particle swarm optimization to solve multi-objective optimization problems [25], and now the MOPSO algorithm has become a very popular method for solving multi-objective optimization problems. As an improved method of particle swarm optimization, MOPSO inherits advantages including simple structure, high efficiency, fast convergence speed [26], and good global search ability [27,28] in particular. When it comes to solving MOPs, because of conflicts between the different goals of MOPs, there is no perfect solution that can meet all the requirements at the same time in most cases. Therefore, the goal of solving an MOP is to find a set of Pareto non-dominated solutions that keeps a balance between the different objectives [29], and the set of all Pareto non-dominated solutions is called the Pareto optimal set [24]. Ultimately, the optimal solution can be selected artificially. MOPSO can effectively search for non-dominated solutions. It is a concise and fast multi-objective optimization method.

**Figure 1:**
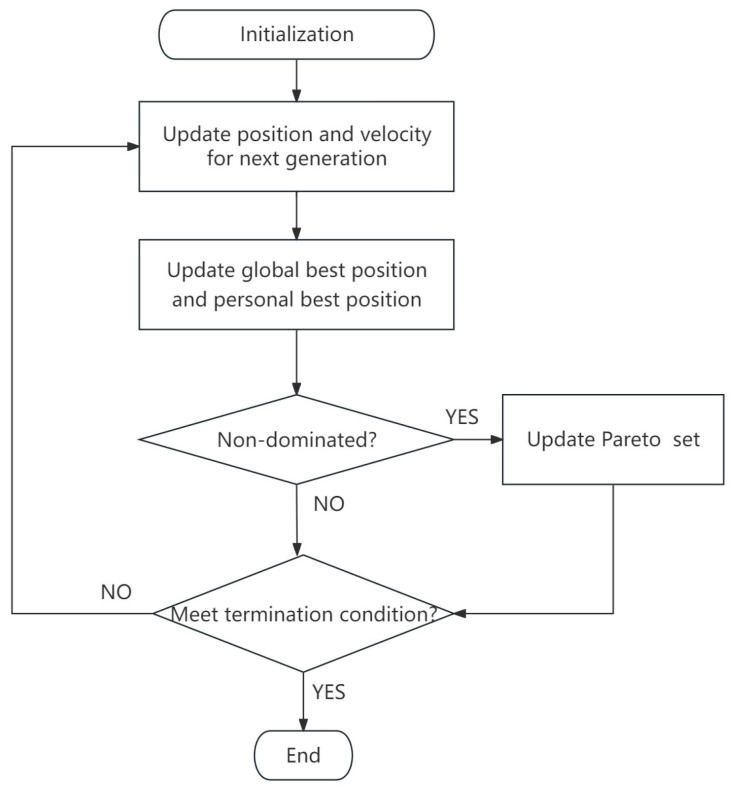
MOPSO flowchart.

The flowchart of the MOPSO algorithm is shown in Figure 1. The first step is to initialize the position and velocity of particles and calculate the global best (*g*_i_) position and personal best (*p*_i_) position corresponding to the initial states of the particles. Each particle *i* with *n* dimensions has the position defined as

(1) $$\vec{x^{i}}=x_{1}^{i},x_{2}^{i},\ldots x_{n}^{i}$$

And a velocity defined as

(2) $$\vec{v^{i}}=v_{1}^{i},v_{2}^{i},\ldots v_{n}^{i}$$

In the process of optimization, particles evolve several generations to find the optimal solution, and the position and velocity of each particle are affected by *p*_i_ and g_i_ when updating the values [30]. In generation *t +* 1, the velocity and position of particle *i* are calculated as follows:

(3) $$v_{j,t+1}^{i}=wv_{j,t}^{i}+c_{1}R_{1}(p_{j,t}^{i}-x_{j,t}^{i})+c_{2}R_{2}(g_{j,t}^{i}-x_{j,t}^{i})$$

(4) $$x_{j,t+1}^{i}=x_{j,t}^{i}+v_{j,t}^{i}$$

where *j* = 1, 2, 3 … *n*. *w* is the inertia weight of the particle, *c*_1_ and *c*_2_ are two positive constants, and *R*_1_ and *R*_2_ are random values in the range [0, 1]. After updating the position and velocity of the particles, calculate new *p^i^* and *g*_i_. If the new optimized solution is a non-dominated solution, it will be stored in the Pareto optimal set, and if the Pareto optimal set happens to be full, priority will be given to the particles in sparse regions and the particles in dense regions will be updated [31]. MOPSO will repeat iteratively until a termination condition is met, for example, a maximum number of generations or no change in the Pareto optimization set. At last, we can select the most effective and balanced global best solution based on our needs.

An inverse-designed passive phase shifter consists of an input waveguide, a functional region, and two output waveguides. The width of input and output waveguides is set to be 300 nm so that they can only support the fundamental mode of TE or TM. The footprint of the functional area is set to 4 μm × 3.2 μm with 20 × 16 pixels. A pixel is square with a size of 200 nm × 200 nm. The pixels composed of silicon are encoded as 1, while those etched with air cylinders are encoded as 0.

Optimizations are carried out in MEEP, and the FDTD method is used to study the performance of the designed structures [32]. A perfectly matched layer (PML) is set at the edge of the simulation area to absorb electromagnetic fields. The silicon layer is 220 nm with a permittivity of ε_Si_ = 11.97 (refractive index 3.46) [13], which is placed on top of a silica buffer layer with a permittivity of ε_Silica_ = 2.10 (refractive index 1.45) [33]. Here, D = 100 nm is chosen as the diameter of the etched air cylinders. A light source is placed at the far end of the input waveguide, and monitors fixed at input and output waveguides are used for capturing the phases and transmitted fluxes, thereby evaluating the performance of devices.

Wavelength modulation is the way by which the passive phase shifters change phase. More precisely, the phase difference between the upper and lower ports should change linearly with wavelength. The FOM of the MOPSO algorithm is defined as follows:

(5) $$a=\alpha_{1}||angle_{up}|-angle_{0}|+\beta_{1}|var_{up}+var_{1}|$$

(6) $$b=\alpha_{2}||angle_{down}|-angle_{0}|+\beta_{2}|var_{down}+var_{2}|$$

(7) $$FOM=\left[\begin{array}{c} a\\ b \end{array}\right]$$

where *angle_up_* and *angle_down_* represent the phases of the upper and lower ports at 1565 nm, respectively. *angle*_0_ is the ideal phase at 1565 nm, which is set to *angle*_0_ = 180° for convenience. That is, if the optimization result is good, the values of *angle_up_* and *angle_down_* should be equal to 180° approximately. In this way, the phase difference between the upper and lower output ports can be approximately equal to 0 at 1565 nm.

The value of phase shift is represented by *var*. *var*_1_ and *var*_2_ are defined as the expected phase shift values of the two output ports in the functional wavelength range, respectively. Similarly, *var_up_* and *var_down_* represent the measured phase shift values of the output ports. It is proven that the phase difference of 2π between two output ports can be achieved in a wavelength range of 30 nm. Therefore, the final wavelength range for the designed device is 1535–1565 nm. When the phase shift values *var*_1_ = 2π and *var*_2_ = 4π, the phase shift difference of 2π can be achieved at 1535 nm.

Additionally, weighting coefficients *α*_1_, *α*_2_, *β*_1_, and *β*_2_ are used for balancing the first and second terms to obtain better optimization results. Keeping the values of FOM terms in the same order of magnitude is helpful in balancing the different parameters of the designed devices in the optimization process.

**Figure 2:**
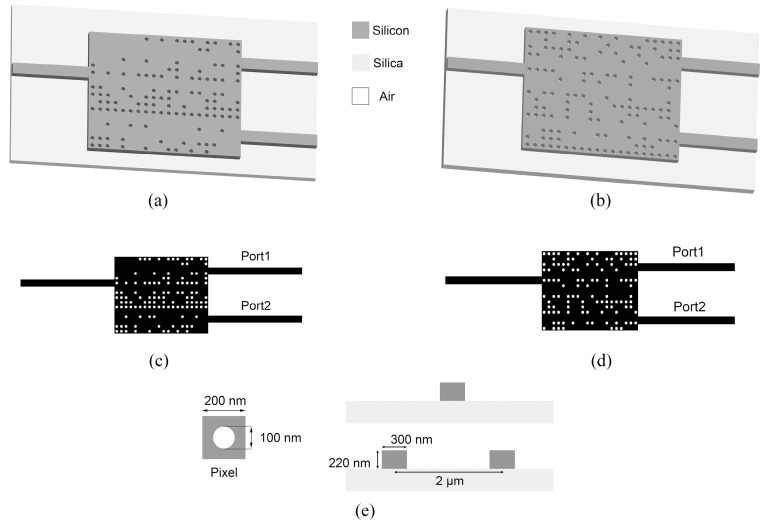
Structures of the passive phase shifters. (**a**) 3−D structural schematic of the TE mode device. (**b**) Top view of the TE mode device. (**c**) 3−D l structural schematic of the TM mode device. (**d**) Top view of the TM mode device. (**e**) Top view of a pixel and side view of the input and output waveguides.

After establishing the simulation model and defining the FOM, the optimized structures of passive phase shifters can be obtained by the MOPSO algorithm, which are plotted in Figure 2. Figure 2a–d show the TE and TM mode phase shifters, respectively. The pixel’s structure and the cross-sections of the input and output waveguides are shown in Figure 2e.

## 3. Results and Discussion

**Figure 3:**
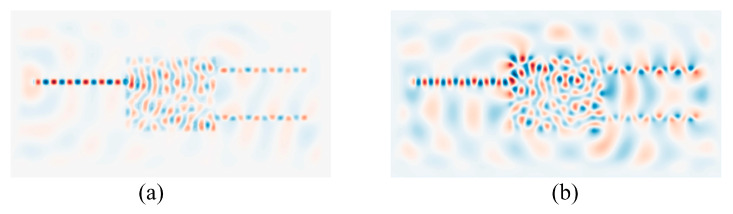
Field distribution of phase shifters for (**a**) TE mode and (**b**)TM mode.

After structure optimization, the finite difference time domain (FDTD) method is used to simulate the optical field distribution. Figure 3a,b show the optical field of the TE and TM mode phase shifters, respectively, at a wavelength of 1560 nm. Due to the asymmetry of the phase shifter structure, light arriving at the two output ports has experienced different transmission distances, resulting in a certain optical path difference between the two output beams. Therefore, a certain phase difference is obtained between the two output beams. Because of the polarization sensitivity of nanostructures [34], the TE and TM phase shifters have different structures and show different field distributions, as shown in Figure 3.

**Figure 4:**
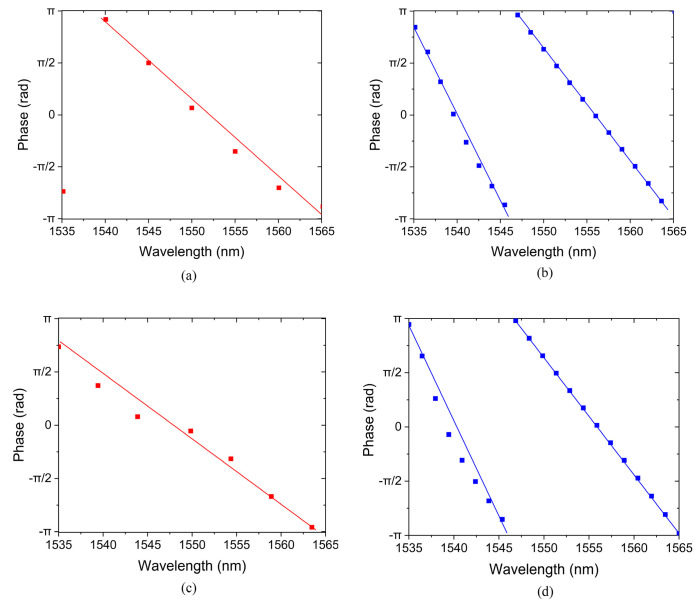
Phase maps of (**a**) Port1 of TE mode device. (**b**) Port2 of TE mode device. (**c**) Port1 of TM mode device. (**d**) Port2 of TM mode device.

For passive phase shifters, the mode coefficients of output modes can be used to compute the phases of the different ports. Figure 4a–d show the phases of the TE and TM mode phase shifters, respectively, where (a) and (c) are the phases output from Port1, while (b) and (d) are from Port2. It can be noticed that for both the TE and TM modes, the phases at Port1 and Port2 are very close to–π at the longest wavelength of 1565 nm, suggesting that the first terms of Equations (5) and (6) are effective in the optimization process of MOPSO. As a result, the phase difference between Port1 and Port2 at 1565 nm is quite small. As the wavelength decreases, the phase at all four output ports increases linearly. According to the second term of Equations (5) and (6), the phase shift at Port1 should be as close to 2π as possible from 1535 nm to 1565 nm, while the phase shift at Port2 should be as close to 4π as possible. As is shown in Figure 4a, as the wavelength decreases from 1565 to 1540 nm, the phase nearly linearly changes from approximately −π to π. When the wavelength further decreases to 1535 nm, the phase value skips to approximately −3π/4, suggesting the beginning of a new cycle. While at Port2, as shown in Figure 4b, as the wavelength changes from 1565 nm to 1547 nm, the phase linearly increases from −π to π at first, and then suddenly skips to −π at 1545 nm, marking the beginning of a new period. As the wavelength further decreases from 1545 to 1535 nm, the phase linearly increases from−π to π. That is, the phase changes by two periods, resulting in a total phase shift value of 4π. Similar results are obtained for the TM mode, as shown in Figure 4c,d. At the longest wavelength of 1565 nm, the phase difference between Port1 and Port2 is close to 0. As the wavelength decreases, the phase difference gradually increases. At the shortest wavelength of 1535 nm, the phase difference reaches approximately 2π.

For the TE mode phase shifter, the phase variation in Port1 and Port2 is 5.83 and 12.08 rad, respectively, resulting in a phase shift difference of 6.26 rad, close to the design goal. For the TM mode phase shifter, the phase shift difference is 6.95 rad. In an OPA, phase difference contributes to beam steering. If the phase difference is 0, the light will not deflect and the beam-steering angle is 0. As the phase difference increases, the light spot in the far field moves from center to edge. When the phase difference increases to π, it will reach the edge of the field of view, where the maximum beam-steering angle can be measured. Then, the observation should continue from the other side of the field of view [12]. While phase difference increases from π to 2π, the light beam steers from the edge to the center in the field of view. When the phase difference reaches 2π, the light spot moves back to the center and the whole field of view is scanned.

**Figure 5:**
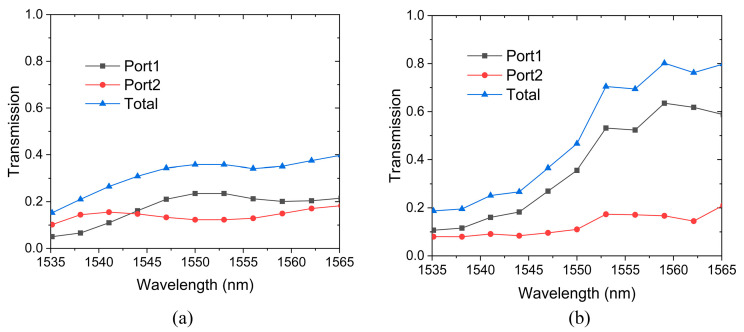
Transmission of phase shifters. (**a**) TE mode. (**b**) TM mode.

Figure 5a,b show the transmission characteristics of phase shifters for the TE and TM modes, respectively. For the TE mode phase shifter, the total transmission fluctuates in the wavelength range, with a minimum value of 15.2% at 1535 nm and a maximum value of 39.8% at 1565 nm. The average transmission is 31.5% in the wavelength range. The transmission of Port1 and Port2 changes in the range of 10−20%, with an average transmittance of 17.3% and 14.2%, respectively. For the TM mode phase shifter, the lowest transmission is 18.7% at the wavelength of 1535 nm. As the wavelength increases, the transmission increases rapidly and reaches a maximum value of 80.2% at 1560 nm. The transmission curve of Port2 is similar to that of the TE shifter, fluctuating in the range of 8.0−21.0% with an average transmission of 12.6%. For Port1, the transmission increases from 10.7% to 58.7% as the wavelength increases. Due to the high transmission of Port 1, the TM phase shifter exhibits excellent transmission performance at long wavelengths.

Currently, the most common passive phase shifting structure is AWG which generates phase difference by extending the length of the waveguide [35,36]. Here, the inverse-designed phase shifter and AWG-based shifter are compared in terms of footprint and transmission.

**Figure 6:**
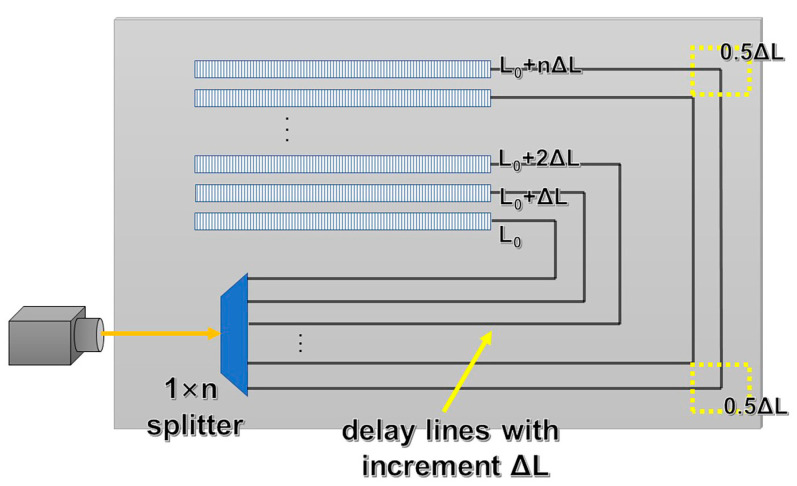
AWG-based phase shifter structure.

A typical AWG structure functioning as a phase shifter is shown in Figure 6 [28]. Input light passes through a 1 × n beam splitter, which divides the input light into *n* beams equally without phase difference. *n* light beams propagate in *n* waveguides, each of which has a C-shaped structure. The outer waveguides are longer than the inner waveguides with a certain length increment between adjacent waveguides. The waveguides are bent into right angles twice, and the length increment at each right angle is defined as 0.5 Δ*L*. Waveguide length difference generates phase difference among the waveguides. Light beams with phase difference are emitted into the air by grating antennas at the end of waveguides, and beam steering is realized by means of wavelength modulation.

The phase difference in AWG between adjacent waveguides is determined as follows [12]:

(8) $$\beta_{n}=n_{eff}\left(\frac{2\pi }{\lambda }\right)n\Delta L$$

where *β_n_* represents the phase difference between *n* waveguides. Here, only phase difference between adjacent waveguides is considered and *n* is set to one. *n_eff_* is the effective refractive index of optical waveguides, *λ* is the wavelength at which light propagates in a vacuum, and Δ*L* represents the length increment between adjacent optical waveguides.

According to Equation (8), to generate a phase difference of 2π in the range of 1535–1565 nm, the waveguide length increment is determined to be Δ*L* = 14.6 μm. For comparison, the footprint of our inverse-designed phase shifter is only 4 μm × 3.2 μm, much smaller than the AWG structure. Due to the complicated nanostructure of the inverse-designed phase shifter, complex diffraction takes place within the functional area, resulting in phase shifting in a tiny area. The extremely small footprint of the inverse-designed phase shifter makes it particularly promising in ultra-compact OPA chips for miniaturized lidars.

**Figure 7:**
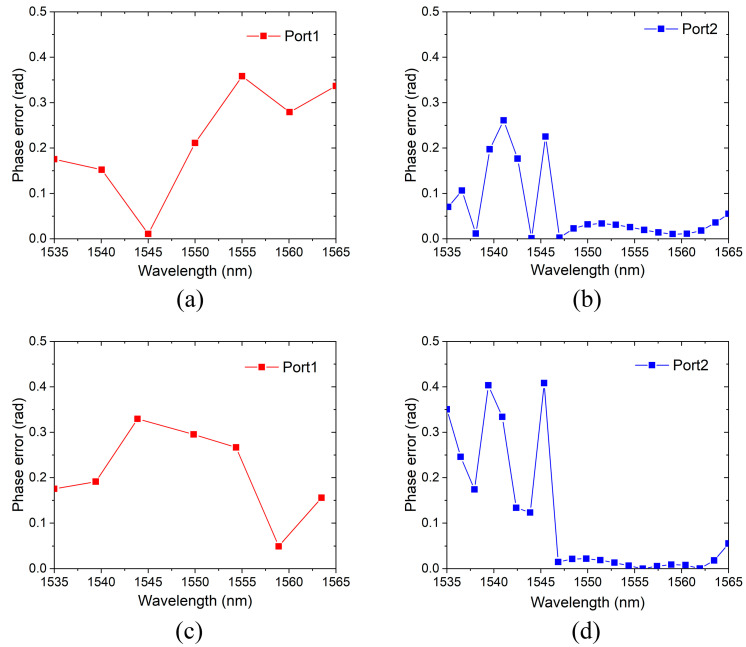
Phase error of inversely designed phase shifters. (**a**) Port1 of TE mode device. (**b**) Port2 of TE mode device. (**c**) Port1 of TM mode device. (**d**) Port2 of TM mode device.

The phase error distribution of the two inversely designed phase shifters is plotted in Figure 7. Figure 7a,b are related to the Port1 and Port2 of the TE mode device, respectively, and Figure 7c,d shows the phase error of Port1 and Port2 of the TM mode devices. The phase differences in the four output ports are all less than 0.5 rad. In contrast, the phase error of AWG varies in the range of 0–4 rad [37]. The results prove that the optimized results are satisfying and the inverse design method is helpful for reducing the phase error of the passive phase shifter.

**Figure 8:**
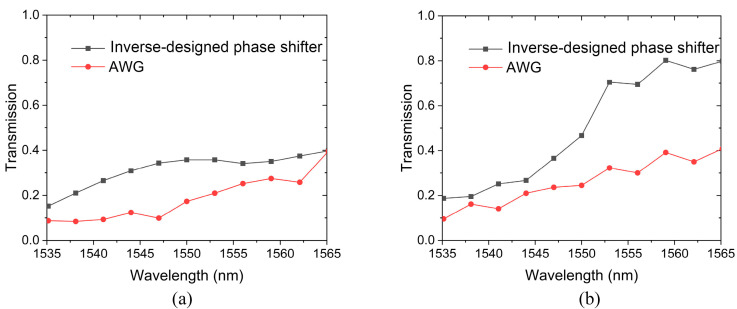
Transmission of inverse-designed phase shifter and AWG counterpart. (**a**) TE mode. (**b**) TM mode.

Figure 8 shows the transmission properties of the inverse-designed phase shifter and its AWG counterpart. It can be seen that for both the TE and TM mode, the transmission of the inverse-designed phase shifter is higher than the AWG counterpart in the whole wavelength range of 1535–1565 nm. For the TE mode, the transmission of the inverse-designed shifter varies from 15.2% to 39.8%, while the AWG counterpart is in the range of 8.4–39.6%, as shown in Figure 8a. For the TM mode, the transmission of the inverse-designed shifter is in the range of 18.7–79.8%, while the AWG counterpart changes from 9.5% to 40.9%. As mentioned above, a relatively large waveguide length increment is required to generate a phase difference in an AWG. To minimize the footprint of AWG, C-shaped waveguides are adopted, whose large bending loss results in low transmission.

## 4. Integrated Beam-Steering Structure

**Figure 9:**
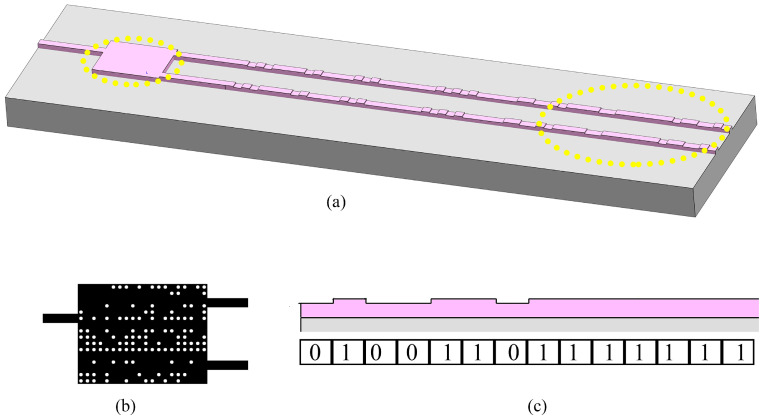
Inverse-designed integrated beam-steering structure for TE mode. (**a**) Three-dimensional scheme of the whole structure. (**b**) Top view of the phase shifter including Si waveguide structure and air holes. (**c**) Side view of the emitter.

**Figure 10:**
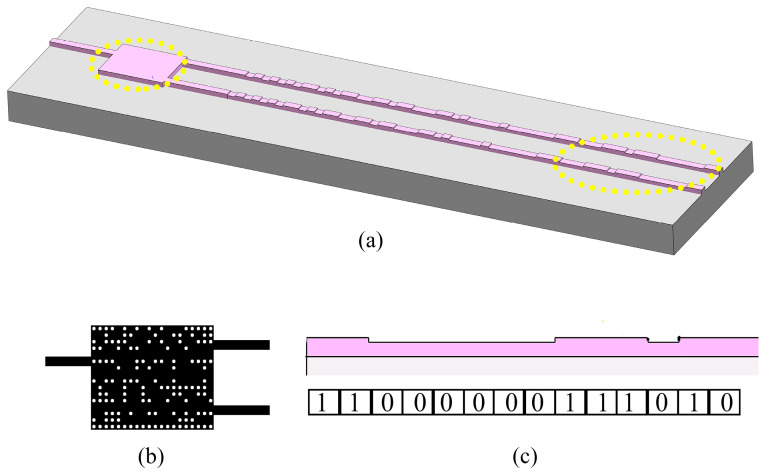
Inverse-designed integrated beam-steering structure for TM mode. (**a**) Three-dimensional scheme of the whole structure. (**b**) Top view of the phase shifter including Si waveguide structure and air holes. (**c**) Side view of the emitter.

By combining the inverse-designed phase shifter in this work and the inverse-designed random-grating emitter previously demonstrated [38], an integrated beam-steering structure is built. Figure 9 and Figure 10 show the beam-steering structure for the TE and TM modes, respectively. The operating principle of the structure is as follows: From the input waveguide, light with a certain wavelength enters the passive phase shifter, where a phase difference is generated between two output beams. As the wavelength changes (e.g., using a wavelength-tunable laser as the light source), the phase difference changes linearly, resulting in far-field beam deflection in the lateral direction after light is emitted into free space by the random gratings.

For the integrated beam-steering structure, the far-field light intensity distribution is used for measuring the scanning angle of the output light beam at different wavelengths. In the lateral direction which is controlled by phase difference, the scanning angle is determined by Equation (9) [38]:

(9) $$\sin(\varphi )=\frac{\lambda \times \Delta \phi }{2\pi (d+W_{wg})}$$

where Δ*ϕ* is the phase shifting difference between output waveguides, *W*_wg_ is the width of waveguides, and *d* represents the distance between adjacent waveguides. The distance between output waveguides *d* is fixed, while Δ*ϕ* changes with the wavelength. Therefore, the beam-steering angle in the lateral direction is affected by the wavelength only.

**Figure 11:**
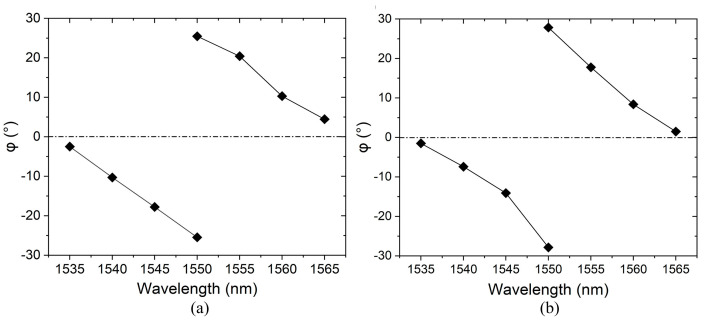
Far−field position of the inverse-designed integrated beam-steering structure. (**a**) TE mode. (**b**) TM mode.

The peak position of light intensity in the far field is shown in Figure 11. *φ* represents the beam-steering angle in the lateral direction of waveguides, which is similar for the TE and TM modes. The first step of measuring beam-steering angle in the *φ* direction is to select one specific beam. At 1565 nm, the beam is near the middle 0° position in the *φ* direction. As the wavelength gets smaller, the beam-steering angle gets larger and the peak position of light intensity moves diagonally towards the edge of the field of view. The beam-steering angle in the *φ* direction reaches its maximum near 1550 nm. Now we focus on a different lobe from the other side of the field of view [12]. As the wavelength continues to increase, the beam-steering angle in the *φ* direction gradually decreases. That is, the peak position of light intensity is diagonally moving from the edge to the center of the field of view. Finally, at the wavelength of 1535 nm, the phase difference reaches approximately 2π and the light beam returns to *φ* = 0°. For the TE and TM modes, a lateral scanning range of ±25.47° and ±27.85° is obtained, respectively, comparable to the other results from active shifters and passive AWG shifters with a much larger footprint [2,12,39,40].

It is still possible to improve some aspects of the beam-steering structure designed in this work. Firstly, there are only two output ports connecting with two waveguide gratings. In order to expand beyond the two output ports to accommodate a larger number of ports, there are two possible methods to be considered in future research. The first way is to change the size of the inverse-designed area and increase the number of output ports. Accordingly, the number of design goals will increase as the number of ports increases. However, this method is limited by the performance of the optimization algorithm and computational resources. Another approach is to explore a cascade structure of inverse-designed passive phase shifters, which is inspired by the imbalanced tree architecture [41]. If the cascade structure can be realized, it would be a promising method to control the phases of many output ports. Multiple output ports have an influence on the performance of beam steering. Increasing the number of output ports will result in a smaller beam width in the lateral direction, which is helpful in improving the resolution of OPA in future work.

## 5. Conclusions

In summary, passive phase shifters for TE and TM polarized modes are inversely designed by the MOPSO algorithm and simulated by FDTD. The phase difference between two output beams is generated after the input light passes through the 4 µm × 3.2 µm rectangular waveguide with random-distributed air-hole arrays, which changes linearly with wavelength. From 1535 nm to 1565 nm, the maximum values of phase shifting difference are 6.26 rad and 6.95 rad for the TE and TM modes, respectively. The average transmission of the TE mode device is 31.5% from 1535 nm to 1565 nm, respectively. For the TM mode, the shifter exhibits an average transmission of 43.3%. The shifters exhibit high transmission with a smaller footprint compared to their AWG counterpart. By combining the inverse-designed phase shifter and random-grating emitter together, an integrated beam-steering structure is built, which shows a large scanning range of ±25.47° and ±27.85° for the TE and TM modes, respectively. This work suggests the great potential of inverse design in the development of ultra-compact high-performance OPA LiDARs.

## Data Availability

The data presented in this study are available upon request from the corresponding author.
